# Do the Interactions Between Type 1 Diabetes and Work Support Self-Management? A Best-Evidence Synthesis

**DOI:** 10.1155/jdr/5523829

**Published:** 2025-05-19

**Authors:** Emma Victoria Shiel, Kim Burton, Steve Hemingway

**Affiliations:** ^1^Department of Psychology, School of Human and Health Sciences, University of Huddersfield, Huddersfield, UK; ^2^Department of Nursing and Midwifery, School of Human and Health Sciences, University of Huddersfield, Huddersfield, UK

**Keywords:** occupational health, self-management, support, Type 1 diabetes, work

## Abstract

Work can be challenging for people with Type 1 diabetes, in part due to difficulties around workplace self-management. What is unclear is the level of accommodation needed, and the type of support required, for effective self-management. To understand the interactions between work and Type 1 diabetes, a best-evidence synthesis of the available grey and peer-reviewed literature was conducted. Twenty-eight articles were included for thematic analysis. Three themes were formulated: (1) How work can be challenging for people with T1D, (2) how work can be beneficial for people with T1D, and (3) influence of policy and legislation. The interactions between Type 1 diabetes and work can hinder self-management. Work-related diabetes distress, concealment, stigma, lack of action space, and work-related intentional hyperglycaemia were reported concerns. Legislation and workplace policy around sickness and disability seem to be relatively inflexible and do not readily accommodate the needs of people with Type 1 diabetes. Conversely, work has acknowledged benefits for health and well-being, indicating a need to facilitate workplace accommodation for people with Type 1 diabetes. Current workplaces do not provide optimal support and accommodation for self-management of Type 1 diabetes. Future research should explore interventions that engage workers in their diabetes management, emphasising individual differences and empowerment. Moving forward, fostering collaborative approaches between the key actors, including managers, human resources, occupational health, and workers with Type 1 diabetes, could be important.

## 1. Introduction

Interest in type 1 diabetes (T1D) has grown due to rising diagnoses and mortality rates worldwide [1]. T1D is an autoimmune disease, where the pancreas fails to produce sufficient insulin to process incoming sugar, resulting in elevated blood sugar levels. Thus, people with T1D must self-administer insulin and regulate blood glucose levels to maintain good health, for which effective self-management plays a crucial role.

“Work” or “employment”—a person's physical or mental efforts to achieve a specific task or goal, usually in exchange for financial reward—is typically good for health providing it is “good” work [2], for example, accommodating a health problem or facilitating self-management. Though self-management support for long-term conditions is recognised, managing T1D at work remains challenging, raising questions about effective strategies [3, 4]. For instance, some roles may not be compatible or lack accommodations, meaning work is prioritised over health and risk is increased [3, 5, 6]

Work is of interest, as it occupies approximately 60% of someone's day [3]. Poor health across this length of time could be detrimental, potentially increasing complications, meaning lower workability. Due to the inconsistency in knowledge transfer, there is a potential disconnect between research findings and policy/legislation. This means that differing relevant information on T1D and work may exist both in academic publications and grey literature, such as professional guidance and policy documents.

To better understand the nuances of T1D and work, an area identified as containing knowledge gaps [6], a review of existing peer-reviewed and grey literature was considered appropriate.

## 2. Materials and Methods

### 2.1. Study Design

A review of literature on T1D and work was conducted using a realist best-evidence synthesis (BES) [7]. BES is distinct from a systematic review in that it lacks certain preconditions and rigid guidelines yet intends to follow a systematic process while allowing tailoring to the specific literature available on the topic, perhaps by combining scientific and grey literature, thus strengthening and broadening the evidence base [7–9]. This review incorporates various BES styles, including those by Slavin [7], Slavin et al., [10], and Burton et al., [11].

Traditional literature reviews, while useful, often overlook diverse knowledge sources. This may overemphasise results from peer-reviewed journals or familiar authors, leading to potential bias [9, 12]. This “file drawer” problem arises when studies with nonsignificant findings remain unpublished and are thus omitted from such reviews [9, 13]. To counter this, a BES was employed for its comprehensive approach in source selection. Including both peer-reviewed and grey literature, a BES ensures a more thorough appraisal of evidence, minimising biases [9, 12]. Additionally, due to the nature of the literature on this subject (including legislative information and diabetes guidance), the inclusion of policy-related grey literature was deemed essential for a complete examination of the topic.

Although less common than traditional systematic reviews and meta-analysis, the BES has found success in the work-health and guidance-policy spheres, often linked with complex policy interventions (e.g., [11, 14, 15]). Thus, this method effectively serves as a knowledge base for government policy in work and health.

### 2.2. Formulating the Research Question

The SPIDER framework was chosen for its effectiveness in mixed-methods research, which will be applied in this review. It serves as an alternative to the PICO tool, developed due to challenges in using PICO for qualitative and mixed-methods research [16, 17].

To reflect the previously identified knowledge gaps [6], the research question is framed as follows: “What existing research and information is there regarding T1D and work, and how does it relate to self-management?”

### 2.3. Eligibility Criteria

The eligibility criteria (Table 1) were chosen to be flexible to ensure as high a retrieval rate as possible.

### 2.4. Selection of Databases (Including Scientific and Grey Literature)

The following literature sources were accessed: Medline, APA PsycINFO, EBSCOhost (including British Education Index, CINAHL Plus, eBook Collection (EBSCOhost), ERIC, and Medline), National Grey Literature Collection, and NHS Evidence. The decision to include these databases reflects a strategic focus on sources most relevant to the research, ensuring comprehensive and multidisciplinary coverage.

**Figure 1:**
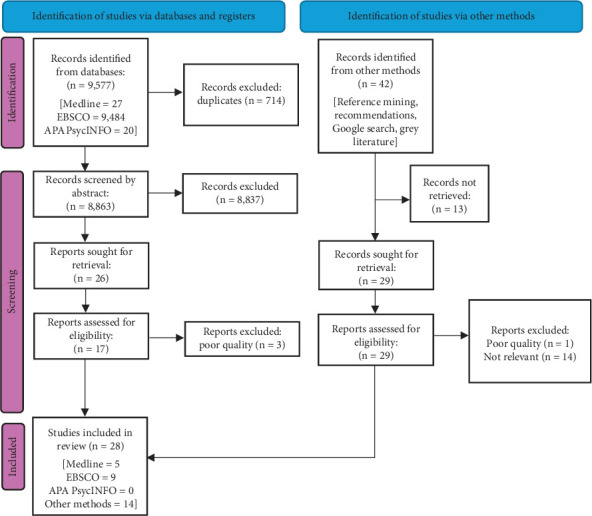
PRISMA flowchart.

Additional articles that impacted the evidence base were included under “other methods” (see Figure 1). These methods, mainly used to identify grey literature, included recommendations, citation mining, and Google searches. Google searches and professional recommendations proved to be the most fruitful.

### 2.5. Development of Search Strategy

An electronic search was carried out in January 2023 and repeated in September 2024. Medline, APA PsycINFO, and EBSCO were systemically searched. Search syntax was constructed using MeSH (Medical Subject Headings) to increase the precision and relevance of results. Search syntax, including Boolean operators, was translated accordingly across databases, for example:

Medline: (“Diabetes Mellitus, Type 1”[Mesh] AND “Work”[Mesh]).

APA PsycINFO: type 1 diabetes mellitus.mp. AND work.mp.

Additional searches using terms “Type 1 Diabetes” and “Work” were made to seek relevant grey literature. Internet search engines and government databases were searched. Contact was made with subject experts who could highlight any missing impactful articles. Reference lists were also scrutinised. All relevant articles passing inclusion criteria were retrieved.

### 2.6. Screening and Study Selection

Retrieved articles were screened by title against the inclusion criteria. Selected articles were then further assessed by abstract, from which the full text of eligible articles was sought; when the final inclusion judgements were made, these were reviewed by a second researcher, and agreement on inclusion was reached.

### 2.7. Data Extraction and Analysis

The extracted data were organised into evidence tables (see Supporting Information(available here)), including information on authors, publication dates, countries, research methods, key features, focal topics (such as T1D, T2D, or other long-term conditions), study types (published or grey literature), and quality. After data extraction, the included articles were expressed thematically using six steps: familiarisation, code formulation, generation of themes, theme review, defining and naming themes, and report formation [18].

### 2.8. Quality Assessment

To ensure accurate representation and eliminate low-quality studies, the MMAT (Mixed Methods Appraisal Tool) was used for peer-reviewed studies. The AACODS Checklist was applied to grey literature for quality assessment. All included articles were graded as “good” quality except for two which were graded as “adequate” [19, 20] (see Supporting Information).

## 3. Results

Twenty-eight articles were included. Half of the articles were retrieved using “other methods” (*n* = 14), and most were published peer-reviewed articles (*n* = 20) rather than grey literature (n = 8). Of the peer-reviewed articles, methods varied between quantitative (*n* = 8), qualitative (*n* = 9), mixed methods (*n* = 2), and reviews (*n* = 1). Geographically, the articles were from the United Kingdom (*n* = 10), the United States (*n* = 2), Denmark (*n* = 4), The Netherlands (*n* = 5), Finland (*n* = 5), Germany (*n* = 1), and the United Nations (*n* = 1). Most studies were on T1D (*n* = 12); however, many included more than one type of diabetes (*n* = 9). Some articles involving other long-term conditions were considered transferable to the knowledge of T1D (*n* = 7) (see Supporting Information).

Thematic analysis of the 28 articles revealed three themes, formulated using the coding, clustering, and reduction methods found in Braun and Clarke's [18] six-step approach: (1) How work can be challenging for people with T1D, (2) how work can be beneficial for people with T1D, and (3) influence of policy and legislation. An outline of the findings, as well as regional comparisons (Tables 2 and 3), is provided.

### 3.1. Theme I: How Work Can Be Challenging for People With T1D

Work, while essential for society, can be challenging for people with T1D if it does not offer support or allow them to manage their condition at work [23–25]. Poor support, high job demands, and limited decision-making latitude are key factors to this, potentially worsening existing health issues and increasing fatigue, leading to less productivity, more sick leave, and possible job loss [20, 25, 26]. Among these, a lack of workplace accommodations for T1D, particularly in shift work, tends to be the biggest contributor [27]. Due to a perceived lack of accommodations, about one in three people with T1D is reported to have “poor” work ability [28].

Diabetes distress refers to the psychological strain of managing diabetes. When compounded by poor working conditions and negative attitudes, this distress can escalate into work-related diabetes distress (WRDD) [23, 29]. Challenges in personal acceptance of the diagnosis and balancing work tasks with diabetes management may exacerbate this, affecting blood glucose, quality of life, and productivity [3, 23, 29, 30].

Among workers with T1D, 49% “sometimes” experience WRDD, while 21% experience it “often,” making WRDD more prevalent than depression among people with T1D [23, 29]. WRDD can be especially detrimental when job demands surpass people's ability to manage tasks alongside fluctuating blood glucose levels. Elevated stress levels have been linked to more frequent and severe hypoglycaemic events, with 63% of workers intentionally not correcting these events so they can remain working [23]. Because of this, some workers with T1D purposely maintain elevated blood glucose to avoid self-management tasks, as well as protecting their colleagues from witnessing the effects of hypoglycaemia [19]. Heavy workloads, limited breaks, lack of job control, and worries about appearing different to societal norms may also contribute to this restriction of self-management [31–33]. Consequently, workers often prioritise work demands over fulfilling diabetes-related needs [32, 33]. Conflicts between health needs and work responsibilities may result in workers beginning to sacrifice self-management to meet work demands or to blend in with their coworkers to avoid appearing different or to avoid damage to their reputation or status [19, 27].

Many participants in these articles felt diabetes was more flexible than work [33]. This often leads workers to skip self-care tasks to avoid interrupting their job, even though it can cause serious health issues [19, 31, 33]. However, this seems more common in younger workers (18–24 years), because they may feel they have “more to lose” due to being early in their career and having not yet established a credible reputation [34].

Generally, workers with diabetes feel less able to negotiate suitable support, compared to their colleagues without diabetes [19], suggesting PWD may be unlikely to ask for help. Thus, to better integrate self-management, workers tend to push T1D into a discreet “side involvement,” managing their condition inconspicuously while continuing to work and maintain a professional appearance (e.g., administering insulin under the table): The term “containment” is used to describe this, where workers stretch their resources to appear both diligent employees and responsible patients [35]. Many participants in the articles tend to feel personally responsible for their diabetes and do not actively seek or expect workplace support [19, 27, 31]. However, although some workers may desire support, concerns of job loss due to diabetes-related challenges can discourage them from disclosing and seeking assistance [27, 35]. A lack of knowledge about diabetes emergencies by colleagues also contributes to some workers feeling there is no value or reward in disclosing [19, 34, 36, 37]. Financial considerations are also relevant since people with chronic illnesses, including T1D, are sometimes considered “more expensive” to employ due to increased sick leave and potential accidents. Thus, disclosing a health condition can be perceived as a barrier to financial stability, and so many people with T1D fail to disclose their condition to the workplace [19, 34].

### 3.2. Theme II: How Work Can Be Beneficial for People With T1D

Work can be beneficial for the health of people with T1D, providing they receive appropriate accommodation [38]. Extensive evidence supports the idea that work contributes to good health, particularly for people with long-term conditions [24, 39]; therefore, avoiding “poor quality” jobs and creating “good” jobs that accommodate people's needs and encourage job retention are becoming increasingly important [39].

Work offers many health benefits: income to acquire essential items, prevention of economic hardship, self-esteem, life satisfaction, and motivation to maintain well-being [32]. These advantages may also encourage effective self-management for people with T1D [27], enabling them to perform to their full potential. For such reasons, encouraging people with T1D to remain in work can be justified to improve quality of life [26]. However, job organisation may need to be varied because it is good “work–life balance” that contributes to better overall health and so a higher likelihood of remaining in work [36].

Being in work also provides regular social interactions and the opportunity for peer support which may empower people with T1D to make positive choices that encourage glycaemic control [27]. Practical assistance from colleagues is particularly helpful, emphasising the value of peers who actively support self-management [27, 32]. However, across the literature, a supportive supervisor or manager appears to have the most influence on how someone feels about their diabetes at work and how effectively they manage it [24, 32, 36]. A “good” manager, someone who organises accommodations for T1D and supports additional needs, is key in helping people to better integrate work and self-management responsibilities [24, 36]. Disclosure plays a crucial role here: Without the disclosure, understanding and addressing workers' needs is unlikely to happen [36, 37].

Nevertheless, people with T1D generally express a desire for independence and ownership over their health [19]; thus, peer support in the form of empowerment and facilitating independence may be important [24]. Weijman et al. [40] supported this, suggesting that improving self-efficacy and confidence through teamwork and achievable targets is a key component of self-management support to overcome challenges. However, workplace interventions that boost workers' self-management confidence remain uncommon [24].

Interestingly, remote working can provide self-management benefits since it grants the privacy and flexibility to freely manage blood glucose levels as well as reducing feelings of stigma and judgement from colleagues [32]. Additionally, remote work increases a person's “action space”—a term describing a person's potential or ability to self-manage—facilitating better glycaemic control [31], leading to improved mental and physical health, meaning higher social participation, self-esteem, and life satisfaction [32]. However, research indicates that different health-affected groups do not equally benefit from identical support, suggesting accommodation should be individualized and continuously adjusted to meet the unique changing needs of each worker [19, 25, 31, 36].

### 3.3. Theme III: Influence of Policy and Legislation

Numerous countries have varying forms of legislation related to employer support for people with health problems and disability. Within UK legislation, the term “reasonable adjustments” is used to describe job and organisational adaptations that UK employers should provide to make jobs more accessible for people with disabilities [22]. The term used in the EU for the same thing is “reasonable accommodation” [41]: Since the United Kingdom uses the term “adjustments” in a legal framework, the more general term “accommodation” is preferred for the purposes of this review when discussing job and organisational changes to accommodate people with T1D. As part of such accommodation, employers might incorporate reduced workloads, time off for sickness and appointments, boundary setting (i.e., declining extra work that might incapacitate them), regular breaks, or access to a calm private space [33, 36, 40, 42]. While some accommodations may not be feasible for a given employer, Hakkarainen et al. [23] suggested that minor work arrangements are adequate for most people with T1D, to ensure that work serves as a motivator for effective self-management rather than a source of fatigue [27, 32].

While the effects of legislation can influence workplace accommodation for people with disabilities, making easier for people with disabilities like T1D, people with diabetes have tended to find legal frameworks to be generic, lacking specific guidance, and unsuitable for their perceived needs [19]. To tackle this, the National Institute for Health and Care Excellence (NICE) guidelines [43] approach to diabetes in work emphasises personalisation, looking specifically at coexisting conditions, and patient needs, whereby each person with T1D is treated individually, rather than as part of a generalised health-affected population. Additionally, NICE [43] suggests a proactive coordinated care plan that is suitable for workers with T1D. Implementing clinical care plans before issues arise is advised to prevent initial complications [36], while NICE [43] states that care plans should be reviewed and updated in accordance with changes in requirements and medical findings.

For incurable conditions (including T1D), adaptive accommodations tailored for regular sickness could help employee retention [44]. The biopsychosocial model of disability, as outlined by Waddell and Aylward [44], provides guidance in this context. An illustrative example is the psychosocial flags framework, originally developed by Kendall et al. [21] for musculoskeletal problems but later adapted for long-term conditions in general, which is a practical system for identifying obstacles to work ability [24]. Hemming's [24] adaptation helps workplaces understand work disability within the context of the individual, the workplace, and the broader social environment. By identifying psychosocial flags, employers can better support employee health through workplace accommodations, social support, and appropriate healthcare.

## 4. Discussion

This review found evidence that balancing work demands with T1D self-management can be challenging. Generally, interactions between T1D and work tend to hinder self-management. Because of this, it has been suggested that the workplace is an ineffective venue for T1D self-management [24], to which WRDD, concealment, stigma, lack of action space, and intentional hyperglycaemia can contribute [23, 29, 30, 37].

However, the benefits of employment generally outweigh the drawbacks of worklessness, such as higher mortality rates, poor health, psychological distress, hospital admissions, and an increased likelihood of long-term illness [2, 23, 29, 30, 37]. Because of this, work is widely accepted as the best form of welfare [2]; thus, accommodating the needs of people with T1D in the workplace is crucial for good health. However, to achieve this, a shift in how employers—and employees—perceive disability, particularly regarding adaptations and support, has been recognised [44] and that need remains unfulfilled [45].

Overall, this review found that employers still misunderstand diabetes and how it might fit alongside someone's job role [36], suggesting a need to increase awareness and education on T1D in some capacity. Key issues include outdated and generic policies, a general lack of understanding about T1D which can make workers hesitant to disclose their condition or seek accommodations, and insufficient understanding and support for diabetes-specific issues like WRDD [23, 29, 31–33, 36].

While typical accommodations may offer some support for workers with T1D, there are additional issues that need to be addressed to fully utilise these provisions. T1D has unique idiosyncrasies, but it also shares with other autoimmune diseases various overlapping challenges (e.g., fatigue, dietary requirements, and distress). This suggests that while some existing measures may benefit those with T1D, there is a strong argument for the need for specific accommodations tailored to T1D that may not currently exist.

Disclosure is an essential consideration when seeking workplace support by those with T1D, as it significantly influences their access to necessary accommodations [19, 34, 36, 37]. Due to ongoing issues with disclosure, future research should explore ways to encourage disclosure or identify methods that do not require full disclosure. Additionally, efforts could focus on helping people with T1D understand the benefits of disclosure, together with guiding managers on effective disclosure practices, given their role in workplace health [24, 32, 36].

Overgaard et al. [33] found that employees often perceive diabetes management as more flexible compared with trying to modify work commitments. This perception allows many people with T1D to temporarily shift their focus between work and health, aiming to be seen as both competent workers and diligent patients when needed. The danger is that this can lead to severe health issues, as at times, workers neglect their health in favour of work responsibilities [33]. Additionally, many people with T1D considered self-management a personal responsibility which discouraged them from seeking or expecting workplace support [19, 27, 31]. To foster positive change, a shift in how workers perceive their health and work responsibilities may be necessary.

A key finding in this review was that not everyone with T1D benefits equally from the same interventions and support [19, 25, 31, 36], so collaborative working could be a core element for developing effective workplace policies. Cooperation between both the worker and employer can help accommodate and reflect the needs of both parties. Wider literature shows collaborative techniques to be transformative for people with long-term conditions, because it avoids previous mistakes of genericism [19] and embeds the person with the health condition within the change making [46], which may increase the likelihood of effective change. Collaboration between key actors, including people with T1D, to actively participate in developing workplace policies, may foster a more inclusive and supportive work environment, benefiting both workers and employers through satisfying each of their needs. Occupational health personnel, human resources, and managers are also likely to play important roles in developing and implementing workplace accommodations [23, 26, 31].

Specific recommendations arising from this review include raising awareness about T1D and associated issues like WRDD, increasing disclosure in the workplace, and helping managers to understand their role in this process, improving general support, addressing feelings of limited decision-making latitude, and enhancing the “work ability” of people with T1D, preventing them from having to choose between their health and their job. Based on these findings, future research should explore changes to workplace health procedures that help people manage their diabetes at work. This could involve tailoring approaches to accommodate individual differences, including more flexible policies and collaborative work-health discussions at the workplace [24, 27, 32, 47].

Finally, while much of the evidence discussed in this review emphasises either preventing future illness or facilitating return to work, it is important for employers to also address the needs of employees who experience frequent and unavoidable illnesses, such as that experienced with T1D. Although T1D is classified as a long-term condition, it is distinct in that people with T1D must undertake daily self-management tasks to ensure their survival, rather than simply maintaining a routine for optimal well-being. Consequently, there is a strong argument that what is needed is the provision of targeted support that addresses the unique challenges faced by people with T1D. Nevertheless, further research is needed to understand the impact of different work environments and roles on people with T1D. It seems that minor work/job modifications may suffice to support self-management for many, yet that will require input from the workplace which, in turn, has implications for local policies and procedures. The current evidence is insufficient to specify precisely what accommodations are needed to effectively support people with T1D at work or, indeed, precisely how they should be implemented. Additionally, selection bias, heterogeneity of methodologies, and the quality of included studies potentially limit the conclusions of this synthesis.

The next research step on this topic might reasonably address these gaps through qualitative data from people with T1D, and from key actors in the workplace, on how workplace support can best be optimized.

## 5. Conclusion

T1D is a health problem that impacts on workability, yet if its self-management at work is accommodated, workability can be maintained and the health promoting benefits of work realised. The existing literature on self-management support is sparse but overall indicates that workplaces often fail to accommodate people with T1D due to outdated policies, lack of understanding, and obstacles to disclosure.

To resolve these issues, our synthesis reveals that employers need a better understanding of T1D and, particularly, the value of supporting its self-management at work. If specific stakeholders (e.g., policymakers, HR leaders, and occupational health professionals) can concentrate their efforts on fostering a supportive social environment which empowers people to excel both as competent employees and effective self-managers, this could drive positive change for this population. But workers with T1D must actively participate by understanding the value of disclosure in obtaining support and recognising the significance of their own actions. The logical implication is that collaborative approaches, where solutions are agreed between the workplace and the worker with T1D, can lead to effective support.

Current generic policies and practices fail to adequately support people with T1D, who need bespoke accommodations for their unique challenges at work. Thus, policy reform and workplace education would be viable calls to action. Arguably, the most urgent area for further exploration is how best to shift employer and employee attitudes, behaviours, and practices. While collaborative approaches are implicated, the challenge for society, employers, and the occupational health community will be one of implementation.

## Figures and Tables

**Table 1:** Inclusion and exclusion criteria.

	**Inclusion criteria**	**Exclusion criteria**
Geographic location	Any	N/A
Date	Any	N/A
Publication type	Any	N/A
Participants	Adults (working age) A diagnosis of T1D (studies based on long-term conditions can be included, subject to transferability)	Studies solely on infant and elderly participants Studies on T2D (studies that partly include T2D in the sample can be included)
Language	English (or translated)	Articles not available in English
Peer review	Peer review not required; however, the quality of the study must satisfy standards of “best evidence” (see the Quality Assessment section)	Poor quality studies
Study design	Any Grey literature, including policy and legislation (relevant to the topic of T1D and work)	N/A
Setting	Any	N/A

**Table 2:** Table of findings.

**Findings**	**Key points**
Theme I. Work challenges for people with T1D	Work environments can hinder self-management due to poor support and high job demands
	Work-related diabetes distress affects a significant portion of workers with T1D, impacting blood glucose, quality of life, and productivity
	Workers may intentionally maintain elevated blood glucose levels or avoid correcting hypoglycaemic events to manage work demands or to avoid appearing different
	“Containment”: Workers discreetly manage their condition to maintain a professional appearance
	Concerns about job loss and lack of awareness among colleagues can discourage disclosure and seeking support

Theme II. Benefits of work for people with T1D	Work provides income, self-esteem, life satisfaction, and motivation for well-being, which may encourage effective self-management
	Supportive supervisors/managers greatly influence how people feel about their diabetes at work and how they manage it
	Remote working can offer privacy and flexibility for managing blood glucose levels and reduce feelings of stigma
	Accommodations should be individualised and adjusted to meet the unique and changing needs of each worker

Theme III. Influence of policy and legislation	Legislation (e.g., Equality Act 2010 in the United Kingdom) mandates “reasonable adjustments” (or “reasonable accommodation” in the EU) to make jobs accessible
	Legal frameworks may be perceived as generic and not tailored to the specific needs of people with diabetes
	NICE guidelines emphasise personalised care plans that are proactive and regularly updated
	The psychosocial flags framework [21] can help identify obstacles to work ability, facilitating workplace accommodations and support

Recommendations arising from this review	Raise awareness and education about T1D in the workplace
	Encourage disclosure and improve support for diabetes-specific issues like work-related diabetes distress
	Promote collaborative approaches involving workers and employers to develop effective workplace policies and individual accommodations
	Address the needs of employees with frequent illnesses and provide targeted support for the unique challenges faced by people with T1D

**Table 3:** Regional comparison of Type 1 diabetes management in the workplace.

**Topic**	**United Kingdom and Ireland**	**Finland**	**Denmark**	**The Netherlands**	**Germany**	**United States**	**International**
Work environment and support	Workplaces have potential to support or hinder diabetes self-management Managers often know little about effects of work environment on self-management Equality Act [22] protects people with diabetes as a disability requiring “reasonable adjustments”	One in three workers with T1D reported poor work ability (twice the general population) High job demands/low job control associated with poor work ability Physical work conditions linked to Work-related diabetes distress	Workers with T1D live in tension between competing logics: “The patient” vs. “the worker” Enabling “action space” for T1D management promotes better self-management	Important facilitators: Disclosure, practical information, social/employer support Key inhibitors: Manager/coworker lack of knowledge, dissatisfaction with occupational health support Lack of social support adds to burden of T1D	Psychosocial factors negatively affect self-management: High workload, poor job control, unhygienic environments, fluctuating temperatures, social norms	Social support and work–life balance associated with excellent work ability Work provides meaning and personal achievement for people with T1D Work conflicts with diabetes self-management demands	UN Convention emphasises equal rights, nondiscrimination, and respect for dignity Focus on full participation and inclusion in society

Disclosure and concealment	No legal obligation to disclose diabetes to employer Concerns about stigma/inappropriate treatment Unlawful to ask about health before offering work	52% disclosed to colleagues, 45% to occupational health, and 28% to line managers 30% concealed from colleagues and 20% from line managers Young adults (18–24) most likely to hide from line managers Concealment associated with feeling like an outsider and neglecting treatment	Diabetes management seen as containment	Disclosure and expressing needs identified as important facilitator for staying at work	Not specifically addressed in German research	Concealment and lack of disclosure common Discrimination and stigma in work context	Not specifically addressed in international documents

Glycaemic control at work	Work stress can make diabetes management difficult Workers tend to prioritise work over diabetes control Shift workers likely to have poorer glucose control and higher HbA1c	Work-related diabetes distress common and can negatively affect glycaemic control Reasons for maintaining inappropriately high glucose levels at work need clarification	Work-related diabetes distress associated with intentional hyperglycaemia Both work-related diabetes distress and problem areas in diabetes linked to intentionally high blood glucose	Employees with high workload more likely to perceive insulin injection as burden The most common coping style was “diabetes avoidance”	Most affected self-management areas: Glucose monitoring, insulin injections, dietary control, hypoglycaemia recognition, healthcare use Work often prioritised over health.	Work linked to poor glycaemic control Participants maintain high glycaemic range to avoid lows and additional breaks	Not specifically addressed in international documents

Vulnerability factors	Young adults find self-management difficult in workplace (time pressures and routine issues)	People aged 18–44 most likely to conceal diagnosis Newly diagnosed people at higher risk	Not specifically addressed in Danish research	Not specifically addressed in Dutch research	Not specifically addressed in German research	Not specifically addressed in US research	Not specifically addressed in international documents

Intervention and policy recommendations	Support needs workplace-specific guidance Confidence for self-management rests on managers supporting flexibility Biopsychosocial model accepted as best framework Better health advice needed for shift workers	Need to evaluate how T1D impacts work ability Potentially retrain/modify job roles for new diagnoses Minor workplace changes could be effective Vocational guidance and occupational health personnel could help	Need for interventions targeting tensions between work and diabetes management Further research needed on work-related diabetes distress Interventions should target well-being of workers with T1D	Support should be available to all workers and tailored to specific needs Prevention of work-related problems should be focus of policy and practice Shared responsibility among all stakeholders Need to address barriers to support	New interventions needed to help workers adequately engage in self-management at work	New intervention/better support system warranted Implementing and evaluating strategies resulting from research	Focus on accessibility, equality of opportunity, and nondiscrimination Acceptance of persons with disabilities as part of human diversity

## Data Availability

The data that supports the findings of this study are available in the Supporting Information of this article.

## Supporting Information

Supporting Information Additional supporting information can be found online in the Supporting Information section. Supporting information (Supp. Materials_Data_Extraction.xlsx) is provided to enhance transparency regarding the information extracted from the studies evaluated for eligibility in the best-evidence synthesis. This document includes details such as author information, key features, reviewer comments, focal topics of each article, sample characteristics, study types, methodologies, and assessments of study quality.
