# Supplementary material for: Do the Interactions Between Type 1 Diabetes and Work Support Self-Management? A Best-Evidence Synthesis
Source: J Diabetes Res. 2025 May 19;2025:5523829. doi: 10.1155/jdr/5523829 (PMC12105885; doi:10.1155/jdr/5523829)
Supplement: Supporting Information — Additional supporting information can be found online in the Supporting Information section. Supporting information (Supp. Materials_Data_Extraction.xlsx) is provided to enhance transparency regarding the information extracted from the studies evaluated for eligibility in the best-evidence synthesis. This document includes details such as author information, key features, reviewer comments, focal topics of each article, sample characteristics, study types, methodologies, and assessments of study quality. [file 5523829.f1.docx]

| **No.** | **Author Information** | **Key Features [Reviewer Comments in Square Brackets]** | **Focal Topic: T1D, T2D, Long Term Condition.** | **Sample** | **Type of Study: Published or Grey Literature?** | **Methods: Qualitative, Quantitative, Review.** | **Study Quality: Poor, Adequate, Good. [Reviewer Comments in Square Brackets** |
| --- | --- | --- | --- | --- | --- | --- | --- |
| 1 | Balfe, Myles; Brugha, Ruairi; Smith, Diarmuid; Sreenan, Seamus; Doyle, Frank; Conroy, Ronan, 2014.  Ireland | Why do young adults with Type 1 diabetes find it difficult to manage diabetes in the workplace?  1. Young adults, particularly those in their mid- to late-twenties, tend to experience self-management difficulties in the workplace. This is usually a result of time pressures and routine issues. 2. Findings indicate the importance of researchers paying attention to older young adults, as well as those making the transition to young adulthood, and to the role that environmental contexts play in young adults diabetes management.  [Rationale for expansive age range] | T1D | 32 young adults with T1D. | Published Study | Qualitative Design: Interviews, Thematic Analysis | 5***** GOOD  [Strong, good quality evidence]. Rated using MMAT. |
| 2 | Bosma, Boot, Schaafsma, and Anema, 2020.  The Netherlands. | Facilitators, barriers and support needs for staying at work with a chronic condition: a focus group study  Key Messages: • Important facilitators for staying at work: (1) Disclosure and expressing needs. (2) Receiving practical information on working with a chronic condition. (3) Social and employer support. • Inhibitors of working for those with chronic conditions: (1) Manager and co-worker lack of knowledge about working with and treatment of chronic conditions. (2) Dissatisfaction with occupational physician support. (3) Absence of support for self-employed workers.  • Support should be available to all workers and tailored to specific needs.  [Rationale given for concentrating solely on T1D, interventions/guidance can then be tailored to specific needs]. | Chronic Conditions [Information is transferable to T1D]. | Workers with one or more chronic conditions (n = 30). Participants included employees and (partially) self-employed workers. | Published Study | Qualitative Design: Focus Group. | 5***** GOOD  [Strong, good quality evidence]. |
| 3 | Bosma, Boot, Snippen, Schaafsma, and Anema, 2021.  The Netherlands. | Supporting employees with chronic conditions to stay at work: perspectives of occupational health professionals and organizational representatives  Key Messages: • Opportunities to optimise support included a shared responsibility of all stakeholders involved. • Prevention of work-related problems should be focused on, certainly with regard to policy and practice. • Preventing work-related problems can be achieved by addressing the identified barriers to provide support. • Recommendations given for occupational physicians and organisational representatives to initiate preventive support at an organisational level and in occupational health care.   [Rationale given for involving stakeholders in the co-production of guidelines/intervention]. | Chronic Conditions [Information is transferable to T1D]. | 16 Occupational Physicians. | Published Study | Qualitative Design: Interviews | 5***** GOOD  [Strong, good quality evidence]. |
| 4 | Cleal, Willaing, Stuckey, and Peyrot, 2019.  USA. | Work matters: Diabetes and work life in the second diabetes attitudes, wishes, and needs (DAWN2) study  Key Messages: • Social support in the work environment is vital. • Work brought meaning to participants lives, indicating the importance of retaining people with T1D in work. • Five overarching themes were found: (1) Work as context for learning about diabetes. (2) Work as an arena for personal achievement and self-identity with diabetes. (3) The demands of work conflict with the demands of diabetes self-care. (4) Discrimination and stigma in the context of work. (5) Social support in the context of work.  [People with T1D were overrepresented, this work is more oriented towards T1D. There are suggestions to further this work: implementing and evaluating the strategies resulting from this study]. | T1D T2D | 328 people with diabetes: 93 (28%) with type 1, 235 (72%) with type 2. | Published Study | Qualitative Design: Open Ended Qualitative Questionnaire. | 4**** GOOD  [Elements from the Mixed-Methods could be more transparent]. |
| 5 | Detaille, Haafkens, Hoekstra, and van Dijk, 2006.  The Netherlands. | What employees with diabetes mellitus need to cope at work: views of employees and health professionals.  Key Messages: • Five ways to cope at work were given:  (1) The ability to accept and cope with diabetes management. (2) Supportive health professionals. (3) Supportive work environment. (4) Work adaptations. (5) Good information.  • Patients felt emotional acceptance of diabetes management and communication with colleagues was most important.  • Professionals felt patient's capacity for self-care was most important.  [More participants with T2D.This study highlights patients capacity for self-care… while work accommodations evidently will aid T1D self-management, perhaps more could be done within hospital care/support to influence people’s behaviour towards their control. Could poor attitudes toward glycaemic control be an external factor to poor work health?]. | T1D T2D | 23 employees with diabetes (46% with T1D, 54% with T2D) and 22 health professionals (GP's, occupational physicians and specialists). All of the health professionals were experts in the field of diabetes care. | Published Study | Qualitative Design: Concept Mapping. | 5***** GOOD  [Strong, good quality evidence]. |
| 6 | Diabetes UK, 2019a.  UK. | Supporting someone with diabetes at work: A Guide for Employers and Colleagues  Key Messages: • People with diabetes should ask for 'reasonable adjustments' at work. Employers may have a legal obligation to adhere to a personalised, flexible, schedule e.g., set break times. • Time off for medical appointments must be adhered to, to support self-management and health. • People with diabetes may be entitled to time off for educational courses under the Equality Act 2010. • Diabetes does not usually stop someone fulfilling work tasks. Blanket bans on employing people with diabetes is unacceptable, except in the armed services which is exempt from the Equality Act 2010.  [While considered grey literature, this article is meaningful, and is useful in discussing work accommodations for T1D]. | T1D T2D | N/A. | Grey Literature. | Guidance. | 4**** GOOD  [Date can only be ascertained through document properties. Concern raised]. |
| 7 | Diabetes UK, 2019b.  UK. | Work and diabetes A guide for people with diabetes  Key Messages: • There is no legal obligation to tell an employer about diabetes. • The Equality Act (2010) makes it unlawful to ask about a person's health before offering work.  • Stress levels at work can make it more difficult to manage diabetes.  [Work can be both good and bad for health, work provides purpose and meaning, but can inhibit self-management and therefore poor health outcomes]. | T1D T2D | N/A. | Grey Literature. | Guidance. | 4**** GOOD  [Date can only be ascertained through document properties. Concern raised]. |
| 8 | Hakkarainen, 2017.  Finland. | Type 1 diabetes and work  Key Messages: • The incidence and prevalence of T1D among working aged people has increased, thus the awareness of T1D should be improved in working life. • One in three of workers with T1D reported poor work ability, twice the corresponding value for the general working population. • WRDD was common with potential to negatively affect glycaemic control.  • Intentional concealment of T1D at work was common, around 50% of workers.  • Newly diagnosed people are higher risk. • Reasons for maintaining inappropriately high glucose levels at work need to be clarified.  • It would be beneficial to find out what kind of support and adjustments are needed and should be clarified how these practices could be best developed and transferred between settings. • Evaluation of how T1D specifically impacts work ability is needed. • Requirement to evaluate if new diagnosis of T1D should allow people to retrain for other jobs or change in the workplace.  [Good direction given for future research; rationale given. Interesting arguments on the scope for retraining or modifying a job role for new diagnosis]. | T1D | 688 Working adults (318 women and 366 men). | Grey Literature. | Doctoral Dissertation. | 5***** GOOD  [Strong, good quality evidence]. |
| 9 | Hakkarainen, Moilanen, Hänninen, Heikkinen, and Räsänen, 2016a.  Finland. | Work ability among Finnish workers with type 1 diabetes  Key Messages: • 1/3 working men and women with T1D had poor work ability. • High job demands/low job control was associated with poor work ability.  • Physical work and low worktime control associated with poor work ability in males - but not females. • Females tended to have a higher HbA1c than males.  [High HbA1c was the only 'diabetes-related' variable associated with poor work ability, suggesting issues to stem from the workplace environment, rather than T1D itself. Requirement for changes in working conditions to better accommodate T1D. It is suggested that sex could also play a part in poor control… It might be productive to look at intersectionality]. | T1D | 2500 people with T1D. | Published Study | Quantitative Design: Questionnaire. | 4**** GOOD  [The response rate of 49%. 201 uncompleted questionnaires from respondents who were excluded from the analysis. Possible potential for nonresponse bias]. |
| 10 | Hakkarainen, Moilanen, Hänninen, Heikkinen, and Räsänen, 2016b.  Finland. | Work-related diabetes distress among Finnish workers with type 1 diabetes: a national cross-sectional survey  Key Messages: • Work-related diabetes distress (WRDD) was found to be common among workers with T1D. • WRDD was linked with problems with physical work conditions, work ability, difficulty in accepting diabetes, job demands, and depressive symptoms. • Some of the factors associated with work-related diabetes distress are modifiable, and some are not. Minor changes to work routine could be very effective. • Vocational guidance and occupational health personnel could potentially help - could better adapt job roles for people with T1D.  [Collaborating with people with diabetes to devise means for adapting work to fit medical requirements was recommended – rationale for current project]. | T1D | 1214 People with T1D, aged 18-64. | Published Study | Quantitative Design: Questionnaire, cross-tabulation, chi-square tests, ANOVA analysis, Spearman correlation coefficients, and structural equation modelling. | 4**** GOOD  [Response rate of 49.3 %, risk of nonresponse bias.]. |
| 11 | Hakkarainen, Moilanen, Hänninen, Räsänen, and Munir, 2017.  Finland. | Disclosure of type 1 diabetes at work among Finnish workers  Key Messages: • 52% of the respondents had disclosed their diabetes to their colleagues, 45% to occupational health personnel and 28% to their line manager. • Receiving social support, having good psychosocial work ability, and relations at work was linked with disclosure to colleagues, line managers and occupational health personnel.  • Opportunity to self-manage diabetes was linked with disclosure to colleagues. • Only 50% of respondents disclosed their Type1 diabetes at work, further research is required into the reasons for and consequences of not disclosing a diagnosis  [Disclosure of T1D is key to self-management, supported by above inferences made toward Hakkarainen (2018), positive social change is key to producing an accommodating environment for those with T1D. Providing a supportive environment could increase disclosure and decrease stigma, therefore improve self-management and glycaemic control at work]. | T1D | 767 working-aged respondents with T1D. | Published Study | Quantitative Design: Cross-Sectional Survey. | 4**** GOOD  [The response rate was below half at 49.3%, only 1013 participants out of 2464 were studied, potential of nonresponse bias.]. |
| 12 | Hakkarainen, Munir, Moilanen, Räsänen, and Hänninen, 2018.  Finland | Concealment of type 1 diabetes at work in Finland: a mixed-method study  Key Messages:  • 30% of wage earners with T1D concealed their diagnosis from general colleagues, 20% from their line manager.  • People aged 18-44 years were most likely to conceal their diabetes. However, those aged 18-24 years were most likely to hide their diabetes from their line managers in particular. • Concealment associated with: not disclosing to family, feeling like an 'outsider', being embarrassed about receiving 'special attention' at work, neglecting treatment.  • Overarching themes were: (1) Being perceived as weak. (2) Job discrimination. (3) Unwanted attention. (4) Being seen as a person who uses their T1D for seeking advantages. (5) Losing privacy.  [There seems to be heightened fear of management among young adults – aligning with past literature that suggests them to be higher risk. Concealment tends to be a product of stigma, leading to poor self-management and health outcomes… Perhaps positive social change is key to producing an accommodating environment for those with T1D]. | T1D | 688 People with T1D. | Published Study | Mixed-Method Design: Interviews and Questionnaire. | 4**** GOOD  [Results from the mixed methods were not integrated. The two methods seem more like two separate studies. However, the interviews add additional value to the survey data. Overall, the Quality of both styles of methods are GOOD]. |
| 13 | Hansen, Cleal, Willaing, and Tjørnhøj-Thomsen, 2018a.  Denmark. | Managing type 1 diabetes in the context of work life: A matter of containment.  Key Messages:  • Working people with T1D live in tension between competing logics linked to diabetes and work: ‘The Patient’ and ‘The Worker’.  • Diabetes management referred to as a matter of containment, i.e., keeping diabetes as a 'side involvement' to maintain focus on other life aspects.  • Further research is needed to advance the theory of containment.  [Recommendations given:  “…developing structural and individual interventions targeting the tension between work life and diabetes management may help mitigate the burden of disease in work life”]. | T1D | 38 People with T1D. | Published Study | Qualitative Design: Interviews. | 5***** GOOD  [Strong, good quality evidence]. |
| 14 | Hansen, Olesen, Browne, Skinner, and Willaing, 2018b.  Denmark. | A call for inclusion of work-related diabetes distress in the spectrum of diabetes management: results from a cross-sectional survey among working people with type 1 diabetes.  Key Messages: • Work-related diabetes distress (WRDD) was distinct from diabetes-related emotional distress. • WRDD was found to be a unique contributor to work ability, quality of life, intentional hyperglycaemia at work, and absenteeism  • Further studies are needed to strengthen the conceptual basis of work-related diabetes distress, explore its clinical usefulness and clarify its risk factors  [Although research into WRDD is still in its infancy, these results are consistently reported in other studies]. | T1D | 1126 working people with type 1 diabetes. | Published Study | Quantitative/Cross-Sectional Design. | 5***** GOOD  [Strong, good quality evidence]. |
| 15 | Hansen, Skinner, Olesen, and Willaing, 2019.  Denmark. | Diabetes distress, intentional hyperglycaemia at work, and glycaemic control among workers with type 1 diabetes.  Key Messages: • Work related diabetes distress was strongly affected by work ability, opportunity to self-manage at work, being treated differently due to T1D, and job demands. • Problem areas in diabetes were affected by identity concern, blame, and judgement.  • Both work related diabetes distress and problem areas in diabetes were associated with intention high blood glucose, and therefore poor glycaemic control.  [A model is used to present some findings; however, it is convoluted. Perhaps a model could be incorporated into the current work: a proposed model and framework to guide self-management support in the workplace]. | T1D | 1,030 working adults with type 1 diabetes. | Published Study | Quantitative Design: Cross-Sectional Survey. | 4**** GOOD  [Participants recruited through health records, does not state if random. Potential for selection bias]. |
| 16 | Hemming, 2020.  UK. | Do workers with long-term health conditions need self-management support at work?  Key Messages: • Identifies support needs specific to the workplace and proposes a model for psychosocial self-management support for long term conditions.  • Workers with long term conditions need workplace self-management support.  • Confidence and empowerment to self-manage rests largely on managers supporting flexibility, leeway, and autonomy. • Guidance is needed to support the principles and practice of self-management.  [Consistent with other studies, good self-management at work being a product of support and understanding, but also mental health – psychosocial aspects worth exploring]. | Long Term Conditions. [Information is transferable to T1D]. | Survey: 736 workers including 327 with mental health, musculoskeletal (MSK) and other long-term conditions. Online Diary: 67 Semi-structured interviews: 15 Narrative interviews: 14 TOTAL: 832 | Grey Literature. | Doctoral Dissertation. | 5***** GOOD  [Strong, good quality evidence]. |
| 17 | Kalleberg, 2016.  UK. | Good jobs, bad jobs  Key Messages: • Job quality is rooted in the philosophy that work is central to addressing a variety of social and individual concerns. • People are more likely to remain in jobs that are 'good quality'.  • Historical background into job quality given (see text).  [Useful text on the background of work, and how work relates to health]. | Work and Health. [Information is transferable to T1D]. | N/A. | Grey Literature. | Book Chapter. | 5***** GOOD  [Strong, good quality evidence]. |
| 18 | Loerbroks, Nguyen, Vu-Eickmann, Krichbaum, Kulzer, Icks, and Angerer, 2018.  Germany. | Psychosocial working conditions and diabetes self-management at work: A qualitative study.  Key Messages: • Psychosocial factors in work negatively affected self-management: high workload, poor job control, unhygienic working environments, working under high/fluctuating temperature, social norms, prioritisation of work over health.  • Most affected areas: glucose monitoring, insulin injections, dietary control, the ability to recognise hypoglycaemia, and health care use. • New interventions help workers to adequately engage in self-management at work.  [Consistent with other studies, interventions are still required. Potential of selection bias. Only employed individuals were recruited meaning those with significant difficulties in work were not included. It would be valuable to hear from both employed and unemployed (due to ill health) to properly investigate this area]. | T1D T2D | 30 Adults with diabetes (19 with T1D, 11 with T2D). | Published Study | Qualitative Design: Interviews. | 5***** GOOD  [Strong, good quality evidence]. |
| 19 | McCarthy, Vorderstrasse, Yan, Portillo, and Dickson, 2021.  USA. | Managing Diabetes in the Workplace.  Key Messages: • Social support and work–life balance was associated with excellent work ability.  • Work is linked to poor glycaemic control. • Work tends to be prioritised over diabetes management.  • Participants tended to maintain high glycaemic range to avoid low blood sugar and additional breaks. • Concealment and lack of disclosure of diabetes was common. • A new intervention/better support system is warranted to improve diabetes management.  [Most participants had T2D and were overweight/obese meaning large elements of this study were irrelevant]. | T1D T2D | 101 Adults with diabetes (13% with T1D, 87% with T2D). | Published Study | Convergent Mixed-Method Design: Questionnaire and Interviews. | 4**** GOOD  [Despite methods seemingly being conducted to a high level, there is no proof to evidence such methods being carried out e.g., an ANOVA was completed but there are no snapshots of this process. The author has to assume]. |
| 20 | National Institute for Health and Care Excellence (NICE), 2015.  UK. | Type 1 diabetes in adults: diagnosis and management  Key Messages: • Offers guidance on diagnosis and self-management.  [Lacks depth, NICE does not offer much value in the scope of work]. | T1D | N/A. | Grey Literature. | Legal Guidance/Legislation. | 5***** GOOD  [Strong, good quality evidence]. |
| 21 | Overgaard, Christensen, and Nexø, 2021.  Denmark. | Conflicting logics in a changing action space: Challenges related to type 1 diabetes in work life.  Key Messages: • People with T1D experience a conflict between two logics linked to diabetes and work. • Conflict can be resolved through shifting work from a main priority to a 'side priority', to better manage diabetes for a short period. • Enabling 'action space' for T1D management at work promotes better self-management.  • Need for interventions targeting the well-being of workers with T1D.  [‘Action space’ is not well covered within the field of T1D self-management and work. Valuable are to contribute towards]. | T1D | 22 Adults with T1D. | Published Study | Qualitative Design: Interviews | 5***** GOOD  [Strong, good quality evidence]. |
| 22 | Ruston, Smith, and Fernando, 2013.  UK. | Diabetes in the workplace-diabetic’s perceptions and experiences of managing their disease at work: a qualitative study.  Key Messages: • Workplaces have the potential to support or hinder diabetes self-management. • Participants felt managers knew little of the effects of work environment on their ability to self-manage their diabetes. • Managerial support was not expected.  • Concerns of stigma/being treated inappropriately. • Work tended to be prioritised over diabetes control.  • Participants felt they had to adapt to their jobs, rather the job adapt to their disease.  • Higher blood glucose was common, thereby higher risk of complications.  [Most participants had T1D. There is requirement to educate and support people with diabetes to disclose their illness and seek support. There are suggestions that those who take part in studies are more likely to adhere to self-management, selection bias must be monitored in current study]. | T1D T2D | 43 People with T1D. | Published Study | Qualitative Design: Interviews. | 3*** ADEQUATE  [Uses constant comparative approach which more suited to grounded theory. Poor methodological choice. No clear links between data sources, collection, analysis, and interpretation]. |
| 23 | The Equality Act, 2010.  UK. | The Equality Act, Chapter 6: Disability  Key Messages: • Diabetes is a protected characteristic at work. • The Equality Act requires the workplace to make ‘reasonable adjustments’ for those with disabilities to prevent discrimination. Examples of such are: (1) Flexibility to step away briefly from your role to treat or manage diabetes. (2) Flexibility over lunch and tea breaks. (3) Providing a private place to inject (this should not be a toilet). (4) Offering ‘sharps’ boxes in the workplace. (5) Providing well stocked hypo kits, modifying job roles. • Diabetes is classed as an 'unseen disability' under the Equality Act. | Disability T1D T2D | N/A. | Grey Literature. | Legislation. | 5***** GOOD  [Strong, good quality evidence]. |
| 24 | United Nations, 2006.  International. | Convention on the Rights of Persons with Disabilities  Key Messages: • Purpose:  (1) To promote, protect, and ensure the full and equal enjoyment of all human rights and fundamental freedoms by all persons with disabilities. (2) To promote respect for their inherent dignity. • Principles:  (1) Respect for inherent dignity, individual autonomy including the freedom to make one’s own choices, and independence of persons. (2) Non-discrimination; Full and effective participation and inclusion in society. (3) Respect for difference and acceptance of persons with disabilities as part of human diversity and humanity. (4) Equality of opportunity. (5) Accessibility. (6) Equality between men and women. (7) Respect for the evolving capacities of children with disabilities and respect for the right of children with disabilities to preserve their identities. | Disability. [Information is transferable to T1D]. | N/A. | Grey Literature. | Convention/Legislation. | 5***** GOOD  [Strong, good quality evidence]. |
| 25 | Waddell and Aylward, 2009.  UK. | Models of sickness and disability: applied to common health problems  Key Messages: • This paper accepts that common health problems are an inevitable part of (working) life and should be accommodated for within work. • Examples of positive prevention techniques:  (1) Positive health at work strategies. (2) Early detection and treatment of mild to moderate symptoms. (3) Accommodation of temporary functional limitations from persistent or recurrent symptoms. (4) Interventions to minimise sickness absence. (5) Promote (early) return to (sustained) work. • The biopsychosocial model, and the ICF are now widely accepted as the best framework for disablement and rehabilitation.  [As accepted by this paper, the lens should not necessarily be about solutions for illness but accommodating for illness]. | Long Term Conditions. [Information is transferable to T1D]. | N/A. | Published Review. | Review | 5***** GOOD  [Strong, good quality evidence]. |
| 26 | Weijman, Ros, Rutten, Schaufeli, Schabracq, and Winnubst, 2003.  The Netherlands. | Fatigue in employees with diabetes: its relation with work characteristics and diabetes related burden  Key Messages: • Both work and diabetes related factors are related to fatigue; lack of support, interactions between diabetes and job demands, and job control contribute significantly.  • Fatigue is further exasperated by lack of social support at work, high job demands and lack of decision latitude, adjusting insulin dosage to circumstances, and more extreme diabetes related symptoms. • Both diabetes and work should be taken into consideration, by supervisors and occupational health staff, in the communication with people with diabetes.  [There is discourse on how much fatigue is caused by work, and how much is caused by the disease itself. Links to Moos and Schaefer (1984) who state that the general illness symptoms are the first task individuals are confronted by, therefore work becomes an added stressor. It would be wise to explore how both work and general attitudes towards T1D could be tackled]. | T1D T2D | 292 Employees aged 30–60 years, with insulin treated diabetes. | Published Study | Quantitative Design: Questionnaires. | 5***** GOOD  [Strong, good quality evidence]. |
| 27 | Weijman, Ros, Rutten, Schaufeli, Schabracq, and Winnubst, 2005.  The Netherlands. | The role of work-related and personal factors in diabetes self-management. Patient education and counselling  Key Messages: • Employees with high workload were more likely to perceive injecting insulin as a burden. • Low social support added to the 'burden' of T1D.  • Most common coping style was 'diabetes avoidance', skipping aspects of self-management.  [This study focuses more on the mental health aspects rather than ‘work’. Biopsychosocial approach might be an interesting lens to use for the current study]. | T1D T2D | 292 Employees aged 30–60 years, with insulin treated diabetes. | Published Study | Quantitative Design: Questionnaire. | 5***** GOOD  [Strong, good quality evidence]. |
| 28 | Young, Waclawski, Young, and Spencer, 2013.  UK. | Control of type 1 diabetes mellitus and shift work.  Key Messages: • Shift workers were likely to have poorer glucose control and higher HbA1c than non-shift workers. • Better health advice is needed for those who work shifts.  [Shift work could be a neglected area due to the unpredictable nature of the working style. Shift work is incompatible with T1D self-management…Should there be accommodations to avoid people with T1D working unpredictable shift patterns to better manage their T1D?]. | T1D | 296 People with T1D. | Published Study | Quantitative Design: Questionnaire | 3*** ADEQUATE  [Twenty-two per cent (296 of 1370 eligible patients) responded. Potential for nonresponse bias. Measures could be better defined, lack of transparency]. |

**References**

1. Balfe, M., Doyle, F., Smith, D., Sreenan, S., Brugha, R., Hevey, D., & Conroy, R. (2013). What’s distressing about having type 1 diabetes? A qualitative study of young adults’ perspectives. BMC endocrine disorders, 13, 1-14.
2. Bosma, A. R., Boot, C. R. L., Schaafsma, F. G., & Anema, J. R. (2020). Facilitators, barriers and support needs for staying at work with a chronic condition: a focus group study. *BMC Public Health*, *20*(1), 1-11.
3. Bosma, A. R., Boot, C. R. L., Snippen, N. C., Schaafsma, F. G., & Anema, J. R. (2021). Supporting employees with chronic conditions to stay at work: perspectives of occupational health professionals and organizational representatives. *BMC Public Health*, *21*(1), 1-13.
4. Cleal, B., Willaing, I., Stuckey, H., & Peyrot, M. (2019). Work matters: Diabetes and worklife in the second diabetes attitudes, wishes and needs (DAWN2) study. *Diabetes Research and Clinical Practice*, *150*, 90-98.
5. Detaille, S. I., Haafkens, J. A., Hoekstra, J. B., & van Dijk, F. J. (2006). What employees with diabetes mellitus need to cope at work: views of employees and health professionals. *Patient Education and Counseling*, *64*(1-3), 183-190.
6. Diabetes UK (2019a). Supporting someone with diabetes at work: A Guide for Employers and Colleagues. Available at: *chrome-extension://efaidnbmnnnibpcajpcglclefindmkaj/https://www.diabetes.org.uk/resources-s3/2019-10/supporting-someone-at-work_v7.pdf*
7. Diabetes UK (2019b). Work and diabetes A guide for people with diabetes. Available at: *chrome-extension://efaidnbmnnnibpcajpcglclefindmkaj/https://www.diabetes.org.uk/resources-s3/2019-10/EmploymentandDiabetes_A5_v7.pdf*
8. Hakkarainen, P. (2017). *Type 1 diabetes and work* (Doctoral dissertation, Itä-Suomen yliopisto).
9. Hakkarainen, P., Moilanen, L., Hänninen, V., Heikkinen, J., & Räsänen, K. (2016a). Work ability among Finnish workers with type 1 diabetes. *Occupational Medicine*, *66*(6), 446-453.
10. Hakkarainen, P., Moilanen, L., Hänninen, V., Heikkinen, J., & Räsänen, K. (2016b). Work-related diabetes distress among Finnish workers with type 1 diabetes: a national cross-sectional survey. *Journal of Occupational Medicine and Toxicology*, *11*, 1-10.
11. Hakkarainen, P., Moilanen, L., Hänninen, V., Räsänen, K., & Munir, F. (2017). Disclosure of type 1 diabetes at work among Finnish workers. *Diabetic Medicine*, *34*(1), 115-119.
12. Hakkarainen, P., Munir, F., Moilanen, L., Räsänen, K., & Hänninen, V. (2018). Concealment of type 1 diabetes at work in Finland: a mixed-method study. *BMJ open*, *8*(1), e019764.
13. Hansen, U. M., Cleal, B., Willaing, I., & Tjørnhøj-Thomsen, T. (2018a). Managing type 1 diabetes in the context of work life: A matter of containment. *Social Science & Medicine*, *219*, 70-77.
14. Hansen, U. M., Olesen, K., Browne, J. L., Skinner, T. C., & Willaing, I. (2018b). A call for inclusion of work-related diabetes distress in the spectrum of diabetes management: results from a cross-sectional survey among working people with type 1 diabetes. *Diabetes Research and Clinical Practice*, *140*, 139-147.
15. Hansen, U. M., Skinner, T., Olesen, K., & Willaing, I. (2019). Diabetes distress, intentional hyperglycemia at work, and glycemic control among workers with type 1 diabetes. *Diabetes Care*, *42*(5), 797-803.
16. Hemming, S. (2020). *Do workers with long-term health conditions need self-management support at work?* (Doctoral dissertation, Loughborough University). https://repository.lboro.ac.uk/articles/thesis/Do_workers_with_long-term_health_conditions_need_self-management_support_at_work_/13325912?file=25673438
17. Kalleberg, A. L. (2016). Good jobs, bad jobs. *The Sage handbook of the sociology of work and employment*, 129-147.
18. Loerbroks, A., Nguyen, X. Q., Vu-Eickmann, P., Krichbaum, M., Kulzer, B., Icks, A., & Angerer, P. (2018). Psychosocial working conditions and diabetes self-management at work: A qualitative study. *Diabetes research and clinical practice*, *140*, 129-138.
19. McCarthy, M., Vorderstrasse, A., Yan, J., Portillo, A., & Dickson, V. V. (2021). Managing diabetes in the workplace. *Workplace Health & Safety*, *69*(5), 216-223.
20. National Institute for Health and Care Excellence [NICE]. (2015). Type 1 diabetes in adults: diagnosis and management. Available at: chrome-extension://efaidnbmnnnibpcajpcglclefindmkaj/https://www.nice.org.uk/guidance/ng17/resources/type-1-diabetes-in-adults-diagnosis-and-management-pdf-1837276469701
21. Overgaard, M., Christensen, U., & Nexø, M. A. (2021). Conflicting logics in a changing action space: Challenges related to type 1 diabetes in work life. *Qualitative Health Research*, *31*(7), 1247-1259.
22. Ruston, A., Smith, A., & Fernando, B. (2013). Diabetes in the workplace-diabetic’s perceptions and experiences of managing their disease at work: a qualitative study. *BMC Public Health*, *13*, 1-10.
23. The Equality Act. (2010). Chapter 6: Disability. Available at: *chrome-extension://efaidnbmnnnibpcajpcglclefindmkaj/https://www.legislation.gov.uk/ukpga/2010/15/pdfs/ukpga_20100015_en.pdf*
24. United Nations. (2006). Convention on the Rights of Persons with Disabilities. Available at: *chrome-extension://efaidnbmnnnibpcajpcglclefindmkaj/https://www.un.org/disabilities/documents/convention/convoptprot-e.pdf*
25. Waddell, G., & Aylward, M. (2009). *Models of sickness and disability: applied to common health problems*. Royal Society of Medicine Press.
26. Weijman, I., Ros, W. J., Rutten, G. E., Schaufeli, W. B., Schabracq, M. J., & Winnubst, J. A. (2003). Fatigue in employees with diabetes: its relation with work characteristics and diabetes related burden. *Occupational and environmental medicine*, *60*(suppl 1), i93-i98.
27. Weijman, I., Ros, W. J., Rutten, G. E., Schaufeli, W. B., Schabracq, M. J., & Winnubst, J. A. (2005). The role of work-related and personal factors in diabetes self-management. *Patient education and counseling*, *59*(1), 87-96.
28. Young, J., Waclawski, E., Young, J. A., & Spencer, J. (2013). Control of type 1 diabetes mellitus and shift work. *Occupational Medicine*, *63*(1), 70-72.
